# Mutated *PET117* causes complex IV deficiency and is associated with neurodevelopmental regression and medulla oblongata lesions

**DOI:** 10.1007/s00439-017-1794-7

**Published:** 2017-04-06

**Authors:** G. H. Renkema, G. Visser, F. Baertling, L. T. Wintjes, V. M. Wolters, J. van Montfrans, G. A. P. de Kort, P. G. J. Nikkels, P. M. van Hasselt, S. N. van der Crabben, R. J. T. Rodenburg

**Affiliations:** 1 Department of Pediatrics, 774 Translational Metabolic Laboratory (TML), Radboud Center for Mitochondrial Medicine (RCMM), Radboud University Medical Center, P.O. Box 9101, 6500 HB Nijmegen, The Netherlands; 2 Department of Metabolic Diseases, Wilhelmina Children’s Hospital, University Medical Center, Utrecht, The Netherlands; 3 Department of General Pediatrics, Neonatology and Pediatric Cardiology, Heinrich-Heine-University, Duesseldorf, Germany; 4 Department of Pediatric Gastroenterology, Wilhelmina Children’s Hospital, University Medical Center, Utrecht, The Netherlands; 5 Department of Pediatric Immunology and Infectious Diseases, Wilhelmina Children’s Hospital, University Medical Center, Utrecht, The Netherlands; 6 Department of Radiology, University Medical Center, Utrecht, The Netherlands; 7 Department of Pathology, University Medical Center, Utrecht, The Netherlands

## Abstract

The genetic basis of the many progressive, multi systemic, mitochondrial diseases that cause a lack of cellular ATP production is heterogeneous, with defects found both in the mitochondrial genome as well as in the nuclear genome. Many different mutations have been found in the genes encoding subunits of the enzyme complexes of the oxidative phosphorylation system. In addition, mutations in genes encoding proteins involved in the assembly of these complexes are known to cause mitochondrial disorders. Here we describe two sisters with a mitochondrial disease characterized by lesions in the medulla oblongata, as demonstrated by brain magnetic resonance imaging, and an isolated complex IV deficiency and reduced levels of individual complex IV subunits. Whole exome sequencing revealed a homozygous nonsense mutation resulting in a premature stop codon in the gene encoding Pet117, a small protein that has previously been predicted to be a complex IV assembly factor. *PET117* has not been identified as a mitochondrial disease gene before. Lentiviral complementation of patient fibroblasts with wild-type *PET117* restored the complex IV deficiency, proving that the gene defect is responsible for the complex IV deficiency in the patients, and indicating a pivotal role of this protein in the proper functioning of complex IV. Although previous studies had suggested a possible role of this protein in the insertion of copper into complex IV, studies in patient fibroblasts could not confirm this. This case presentation thus implicates mutations in *PET117* as a novel cause of mitochondrial disease.

**Electronic supplementary material:**

The online version of this article (doi:10.1007/s00439-017-1794-7) contains supplementary material, which is available to authorized users.

## Introduction

Cellular respiration involves a series of biochemical reactions by which nutrients are oxidized. The energy that is released from these reactions can be used to convert ADP into ATP, the energy currency of the cell. This metabolic pathway takes place in the mitochondria and is executed by the concerted action of several large protein complexes (complex I–IV), called the electron transport chain (ETC), and results in a proton gradient over the mitochondrial inner membrane. This proton gradient is used by complex V, ATP synthase, to drive the production of ATP. The actual use of oxygen takes place at the level of complex IV (cytochrome c oxidase), the final complex of the ETC.

Although the number of patients with a mitochondrial defect is relatively high—with an estimated incidence of at least 1:5000 (Sanderson et al. 2006)—the group is exceptionally heterogeneous, both with regard to the clinical symptoms, as well as to the underlying genetic defects. In general these diseases are progressive, multi system disorders, and usually involve a lack of cellular ATP production (Chinnery and Hudson 2013).

Complex IV deficiency is the second most abundant isolated ETC complex deficiency occurring in 19–27% of all mitochondrial patients (Debray et al. 2007; Scaglia et al. 2004) and in the majority of cases results in a severe, often fatal, infantile disease. Mammalian complex IV consists of 14 polypeptide chains (Balsa et al. 2012; Pitceathly et al. 2013) of which three are encoded by the mitochondrial DNA (mtDNA) and 11 by the nuclear genome (nDNA)). In addition, complex IV contains two heme groups, two cytochromes, and two copper centers. Only a small proportion of the complex IV deficiencies is caused by mutations in the genes encoding structural complex IV subunits. In rare cases, heteroplasmic mutations are found in the three mtDNA encoded subunits (COXI, COXII, and COXIII) (reviewed in Schon et al. 2012; Shoubridge 2001). More recently, also mutations in some of the nDNA encoded structural subunits have been found associated with disease. These are COX7B (Indrieri et al. 2012), COX6B1 (Abdulhag et al. 2015; Massa et al. 2008), NDUFA4 (Balsa et al. 2012; Pitceathly et al. 2013), COX4-2 (Shteyer et al. 2009), COX6A1 (Tamiya et al. 2014), and COX8A (Hallmann et al. 2016).

Most mutations that result in an isolated complex IV deficiency have been found in genes encoding proteins involved in the many different aspects of the construction of an active complex IV, such as the transcription, translation, and assembly of the subunits, and biosynthesis of heme *a* and the Cu_A_ site of COX2. These genes include *COX10*, *COX14*, *COX15*, *COX20*, *COA5*, *COA6*, *SCO1*, *SCO2*, *SURF1*, *TACO1*, *FASTKD2*, *LRPPRC*, *PET100* (reviewed in Dennerlein and Rehling 2015; Kadenbach and Huttemann 2015; Ng and Turnbull 2016), and most recently *COA3* (Ostergaard et al. 2015).

The number of genes encoding assembly factors that are potential mitochondrial disease genes is potentially much larger than the number that has now been identified. Yeast studies have identified more than 34 different complementation groups for nuclear factors involved in complex IV biogenesis (McEwen et al. 1986). These are a subset of the so-called nuclear *Pet* (*petite*) genes. Yeast carrying a mutation in a *Pet* gene has lost the ability to utilize non-fermentable, but not fermentable, carbon sources (McEwen et al. 1986; Tzagoloff and Dieckmann 1990). The homology of the yeast assembly factors with their human counterparts is generally low, making identifications of the human complex IV assembly factors by orthology screening rather difficult. Using ortho-profile, an iterative orthology prediction method, Szklarczyk et al. (2012) predicted several genes encoding potential human complex IV assembly factors. Of some of these their mitochondrial localization had not even been correctly annotated before. On the basis of these predictions, a pathogenic defect in C2orf64/COA5 was detected in a family with fatal cardiomyopathy and complex IV deficiency (Huigsloot et al. 2011).

Since these predictions were made, the involvement of several of these putative assembly factors in complex IV function has been demonstrated. These are C12orf62/COX14 (Weraarpachai et al. 2012), PET100 (Lim et al. 2014; Olahova et al. 2015), FAM36A/COX20 (Doss et al. 2014; Szklarczyk et al. 2013), and COA3 (Ostergaard et al. 2015), and possibly also PET309/PTCD1, in which a genetic variant was identified by whole exome sequencing of a complex IV deficient patient, although this gene variant has not been functionally characterized yet (Taylor et al. 2014).

Here we describe two sisters born from consanguineous parents who presented with signs of mitochondrial disease. Whole exome sequencing revealed a homozygous mutation in *PET117*, a putative complex IV assembly factor (McEwen et al. 1993; Szklarczyk et al. 2012) not previously identified as a mitochondrial disease gene.

## Materials and methods

### Cell culture

Fibroblasts were cultured using standard procedures in M199 medium (Gibco) supplemented with 10% fetal calf serum (FCS, PAA), and 1% penicillin/streptomycin (Gibco) at 37 °C with 5% CO_2_. Cell lines used were primary fibroblasts, from the patients described here as well as from controls (C).

293FT cells (Invitrogen, Breda, The Netherlands) were grown in Dulbecco’s Modified Eagle’s Medium (DMEM) containing 4.5 g/L glucose, 10% FCS, 4 mM l-glutamine, 1 mM sodium pyruvate, 0.1 mM MEM non-essential amino acids, 1% penicillin/streptomycin, and 500 μg/ml geneticin (G418). During the transfections for lentivirus production, the medium did not contain penicillin/streptomycin and geneticin. This study adhered to the Declaration of Helsinki and written informed consent was obtained from each individual or their parents.

### Enzyme activity measurements

The activities of the separate respiratory chain complexes, citrate synthase (CS), and total protein in fibroblasts and muscle biopsies were measured spectrophotometrically as described before (Janssen et al. 2003; Rodenburg 2011) as part of our routine diagnostics. Measurements were only accepted when each of the duplicate values was within a 10% range of their average. Respiratory chain enzyme activities were normalized on the basis of citrate synthase activity.

### Lysates, electrophoresis, and western blotting

Fibroblasts cell pellets were processed for the analysis of mitochondrial complexes by Blue Native (BN) PAGE as described (Nijtmans et al. 2002). Part of these mitochondrial protein complexes were further processed for SDS-PAGE by adding SDS-PAGE sample buffer. Protein concentrations were measured using a Micro BCA protein assay kit (Thermo Scientific). Mitochondrial extracts were separated on 5–15% BN-PAGE gels (Nijtmans et al. 2002), or on 10% SDS-PAGE.

Antisera used were anti-V5 (Invitrogen), anti-COX-I (Abcam), anti-COX-II (Invitrogen), anti-COX-IV (Abcam), anti-Porin/VDAC (Mitosciences), anti-CI (NDUFA9) (Abcam), anti-CII (SDHA) (Abcam), and anti-CIII (UCCRC2) (Abcam).

### WES analysis and Sanger sequencing

Whole exome sequencing and data analysis were performed essentially as described before (Neveling et al. 2013; Wortmann et al. 2015). Exome enrichment was performed using the SureSelect Human All Exon 50 Mb Kit (Agilent, Santa Clara, CA, USA). An in-house developed graphical user interface was used for the data visualization and filtering of variants. Several filtering steps were performed on the initial datasets of variants to exclude common polymorphisms (using dbSNP v.137, EVS (http://evs.gs.washington.edu/EVS), ExAC (http://exac.broadinstitute.org/), and an in-house database) and include only exonic or splice site and non-synonymous mutations. For the clinical interpretation of variants, a routine pipeline was applied to predict the mutation impact at the protein level, which includes the prediction tools SIFT, Polyphen-2, and AlignGVGD (Adzhubei et al. 2010; Ng and Henikoff 2001; Tavtigian et al. 2006). In addition, genomic conservation as assessed by PhyloP scoring was included in the interpretation of variants (Gilissen et al. 2012; Pollard et al. 2010). We also included database searches into possible protein functions, disease associations and tissue distribution.

The most promising variant was validated by Sanger sequencing in the probands, healthy siblings, and in the parents using the primer pair PET117_exon2_ Fa: tgtaaaacgacggccagtTGGATTTGAATGGCACAAGGATGG and PET117_ exon2_ Ra: caggaaacagctatgaccCTACTAAGCTACTCTCCATCAAC.

### Copper treatment

For copper treatment of patient fibroblasts, a previously published protocol was applied (Salviati et al. 2002) with slight modifications. First, a stock solution of 1000 µM CuCl_2_ was prepared by dissolving CuCl_2_ (Sigma; 459097) in M199 medium. Final concentrations of CuCl_2_ were obtained by further diluting the CuCl_2_ stock solution in M199 medium.

### Lentiviral complementation

Cloning of the constructs and lentiviral transductions were performed as described before (Renkema et al. 2015). In short, a Gateway vector for *PET117* without stop codon (gift from Leo Nijtmans) was recombined with the pLenti6.2V5-DEST destination vector (Invitrogen) using the Gateway technology (Invitrogen).

The resulting pLenti6.2-*PET117*-*V5* or the control construct pLenti6.2-*AcGFP*-*V5* was used to produce viruses in HEK 293FT cells. Infections were performed on fibroblasts in 25 cm^2^ flasks with 1 ml of virus containing supernatant in the presence of 6 µg/ml polybrene (Sigma). Twenty-four hours after infection the medium was refreshed and 48 h after transduction the selection of transduced cells was started with 2 µg/ml blasticidin (InvivoGen). Cells were selected for 14 days, in which time the mock infected cells (without virus) died. Blasticidin resistant cells were used for biochemical analysis.

## Results

### Patient description

#### Subject 1

**Fig. 1:**
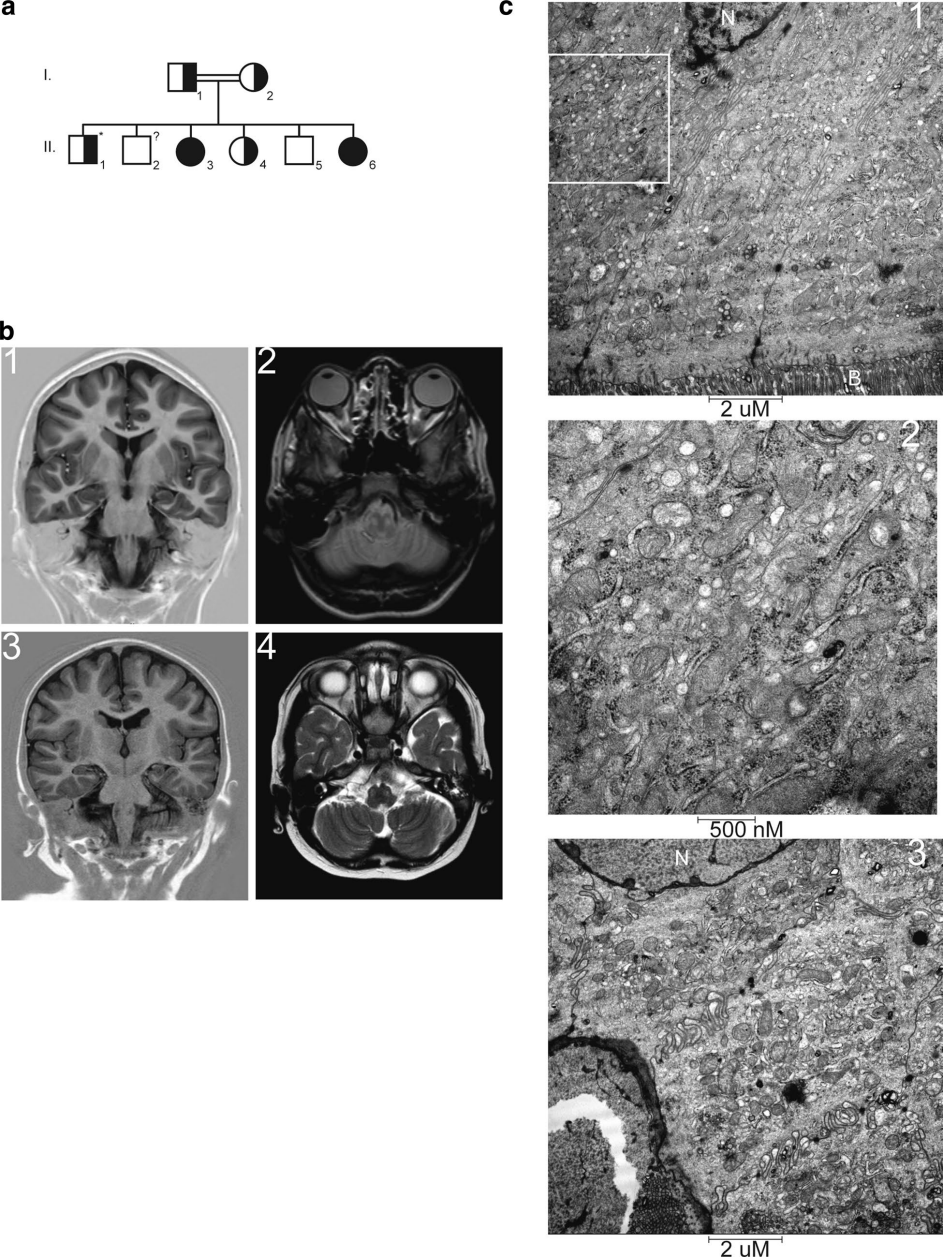
Family tree and brain magnetic resonance imaging (MRI) of the affected siblings. **a** Schematic representation of the family tree. Individual II-2 was lost to follow-up and could, therefore, not be genetically tested. This person is without symptoms. The *asterisk* marks individual II-1 who has intellectual and motor regression, macrocephaly and adiposity but has no indications of mitochondrial disease and his symptoms are assumed to be unrelated to his *PET117* carrier status. Individuals II-3 and II-6 are subjects 1 and 2, respectively. **b1** Inversion recovery (IR) MRI, and **b2** T2 weighted MRI images are of subject 1. **b3** IR MRI image, and **b4** T2 MRI image of subject 2. These images show normal myelination, normal gyral pattern, and normal corpus callosum. Hyperintense areas are noted on the T2 image at the site of the medulla oblongata. **c** Electron microscopic analysis of the ileum of subject 2. Increased numbers of mitochondria were noted on the apical (**c1**, **c2** magnification of the indicated region of c1) and the basal (**c3**) region of the enterocytes. *N* nucleus, *B* brush border

This patient is the third of six children (individual II-3, Fig. 1, Panel a) born from healthy, second degree consanguineous (mothers of the parents are sisters) Moroccan parents. She was born at term and had a normal start. From the age of 2 years an abnormal motor development was noted. Speech and motor skills slowly regressed from 10 years of age onwards, and she now (at the age of 19 years) only speaks some words. She was examined at the age of 15.6 years, at the time when her younger sister (subject 2, see below) showed regression in development. At examination she had a normal growth with normocephaly and no dysmorphic features. On neurological evaluation, pyramidal signs with positive Babinski’s, bradykinesia and hypokinesia were noted. An MRI of her brain showed abnormal lesions on the T2 image of the medulla oblongata (Fig. 1, Panels b1 and b2). Extensive ophthalmological examination showed no pathology (light reflex symmetrical, stereo vision according Lang 3× positive, eye movements normal, funduscopy: papil and macula normal aspect, central minimal tortuous vessels).

Initial metabolic investigation in subject 1 at the age of 13 revealed no abnormalities, apart from a mild elevated plasma glycine levels (375, highest reference value 330 μmol/l). Four years later more signs of mitochondrial involvement could be noted. In urine, mildly elevated excretion of lactate (159; highest reference value 131 mmol/mol), creatine (267; highest reference value 244 mmol/mol), and guanidinoacetate (107; highest reference value 78 mmol/mol). In plasma, glycine was still mildly elevated (365 μmol/l), with normal CSF/plasma ratio. In addition, lactate levels were elevated (3.0, highest reference value 2.0 mmol/l). Other investigations were normal, including chitotriosidase enzyme activity and transferring isoelectrofocussing. In CSF, elevated levels of alanine (41 μmol/l; highest reference value 29,7) and lactate (3.1; highest reference value 2.1 mmol/l) were detected, while other investigations were normal, including 5-methyltetrahydrofolate, 5-hydroxyindolazijnzuur, homovanillic acid, ratio HVA/5HIAA, and 3-*O*-methyldopamine.

#### Subject 2

Subject 2 (individual II-6, Fig. 1, Panel a) is the youngest sister of subject 1 and is currently (2017) 8 years of age. At the age of 20 months she presented for the first time with an episode with fever and diarrhea also with edema and ascites due to protein losing enteropathy (PLE). She was frequently admitted because of recurrent respiratory infections requiring oxygen and intravenous antibiotic treatment. During viral infections, neutropenia was noted. Additional investigations revealed hypogammaglobulinemia with IgG1 and IgG2 subclass deficiency.

Her general development was delayed from birth. From the age of 6 months she suffered from recurring infections and since then her motor development progressed slowed down. Initially this was attributed to her frequent illnesses and hospital admissions. Milestones were all reached at a slightly later age than normal. She was able to walk unaided at 2½ years. Her exercise tolerance was always lower than her healthy peers. An MRI of the brain at the age of 4 years showed no abnormalities. Motor evaluation at the age of 5 years showed a motor development of a 4-year-old. She has normal facial mimicry, no paresis, and no ataxia. BSID-III (American norm) at the age of 40 months revealed a developmental age of 27 months (index 74–90). Wechsler Preschool and Primary Scale of Intelligence—third edition (WPPSI-III-NL), at the age of 4 years and 11 months was far below average scores and reference ages, varying from <2; 7 years to <4; 10 years. Currently, she cannot walk stairs anymore, and can only walk unaided for short distances, and has a similar movement disorder as her affected sibling. She is able to speak but her memory declines. An ophtalmological examination showed no pathology, as in subject 1. The cause of the progressive PLE is as yet unknown, despite extensive investigations, including duodenoscopy, colonoscopy, double balloon enteroscopy, MRI enterography, nuclear albumin scan, laparoscopy, and full thickness biopsy of the ileum and repeat ultrasounds. She initially was treated with extensive immunosuppressive medication and giving supportive therapy consisting of ciproxin and weekly albumin transfusions, as well as intravenous immunoglobulins transfusions a few times per year. The only effective treatment was high-dose prednisone, temporarily reducing her need for albumin transfusions from twice a week to once every 6 weeks. This effect did not last and a partial ileum resection was performed twice, at the age of 4 and 7 years due to severe stenosis of the inflamed ileum. Histological examinations of the ileum revealed diffuse erosions and ulcerations, and (only in the second resection) also arteriovenous malformations in the submucosa of unknown origin. Electron microscopy of the ileum showed increased numbers of mitochondria, both on the apical site (Fig. 1, Panels c1 and c2) as well as on the basal site (Panel c3) of the enterocytes. An MRI of the brain at the age of 6 years revealed two abnormal lesions on the T2 image of the medulla oblongata, identical to those found in subject 1 (Fig. 1, Panels b3 and b4). Based on the MRI findings and the clinical features showing neurodevelopmental regression, a mitochondrial disorder was suspected. Metabolic investigations at age 1 year showed no abnormalities in urine, except for excretion of dicarbonic acids probably as result of MCT containing feeding. All other investigations in urine and blood were normal, including lactate, amino acids, acylcarnitine profile. At the age of 5 years, elevated lactate was noted (3.0 mmol/l). In addition, plasma levels of alanine (1465; highest reference value 600 µmol/l) and glycine (417 μmol/l) were observed, with an increased alanine/lysine ratio (6.4; highest reference value 3.0) and alanine:(phenylalanine + tyrosine) ratio (6.6; highest reference value 4.0).

### Biochemical characterization of subjects 1 and 2

Analysis of the OXPHOS complex activities in muscle biopsies from the patients showed an isolated complex IV deficiency as compared to control samples. The activities of the other enzyme complexes were all within the reference range (Table 1).

**Table 1:** Isolated complex IV deficiency in muscle biopsies of subject 1 and subject 2

	CI	CII	CIII	CIV	CV	CS
Subject 1	298	456	947	**123**	719	475
Subject 2	324	412	721	**103**	809	283
Reference range	163–599	335–888	570–1383	288–954	193–819	151-449

Activities of all complex activities are expressed as milliunits per unit citrate synthase (mU/U CS). The activity of CS is expressed as milliunits per milligram protein (mU/mg). Respiratory chain complex activities below the reference range are indicated in bold

**Fig. 2:**
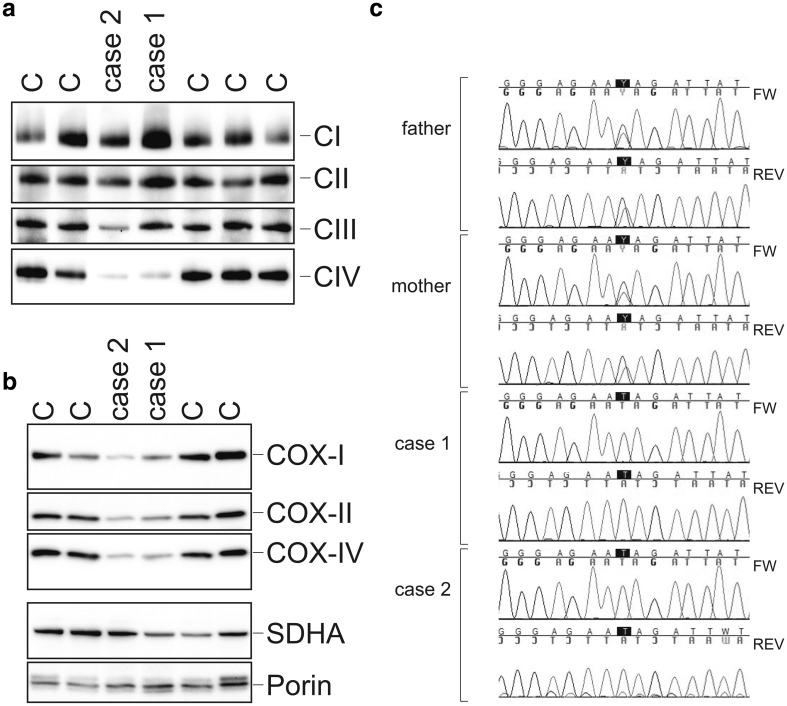
Patients have reduced levels of complex IV, reduced levels of complex IV subunits, and mutations in *PET117*. **a** Blue native electrophoreses and western blot analysis of the patient cell lines compared to three different control cell lines. Blots were probed for complex I (NDUFA9), complex II (SDHA), complex III (UQCRC2), and complex IV (COX-IV). **b** SDS-PAGE separation of fibroblast extracts of the two patient cell lines compared to four different control cell lines. Western blots were probed for complex IV subunits, COX-I, COX-II, and COX-IV. Antisera against CII (SDHA) and Porin were used as loading controls. **c** Sanger sequencing of DNA of the patients as well as the parents confirmed the presence of the c.172C>T mutation. *FW* forward sequence, *REV* reverse sequence

**Fig. 3:**
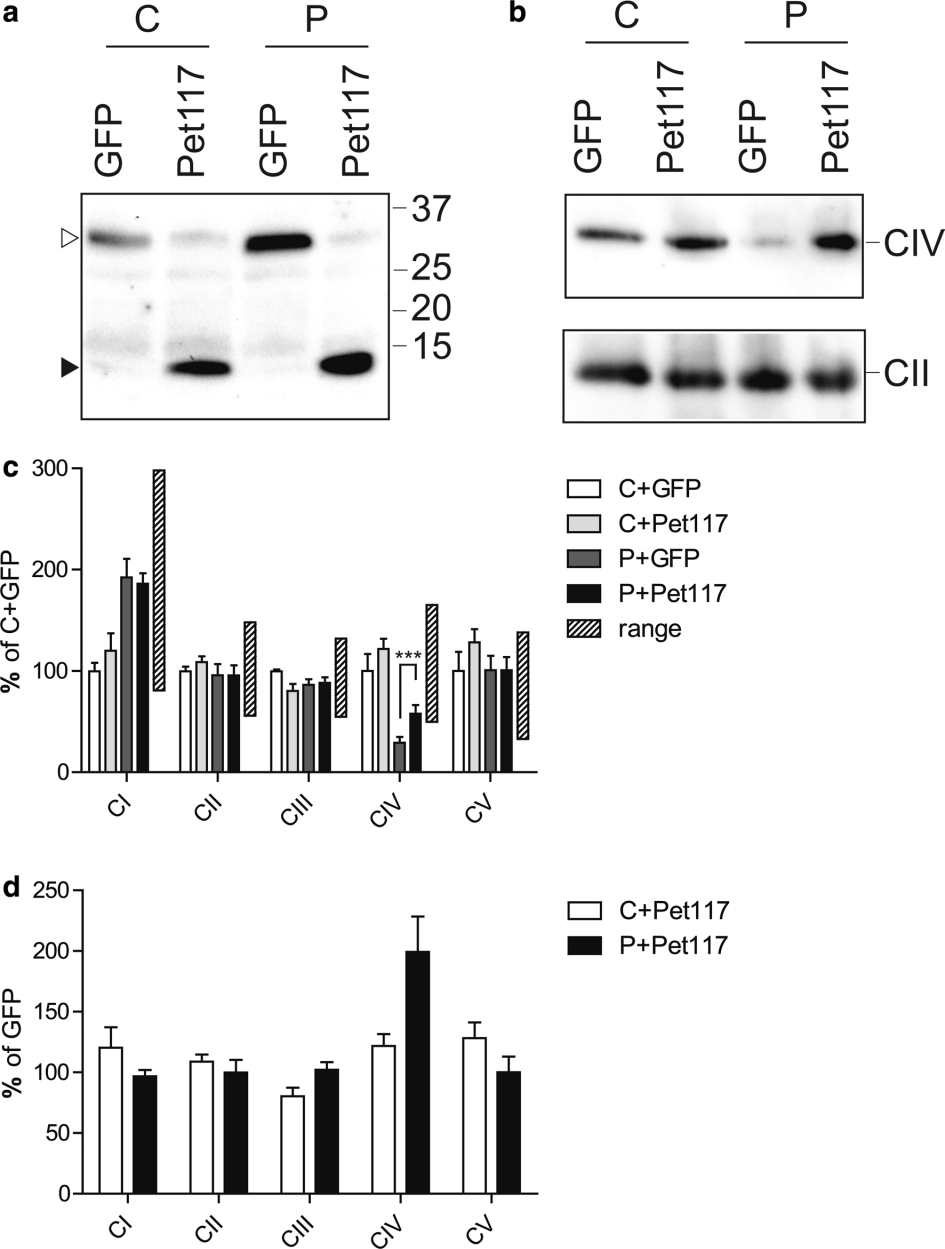
Complementation of fibroblasts with wild-type *PET117* restores complex IV activity. Patient subject 1 (*P*) and control (*C*) fibroblasts were transduced with lentiviruses carrying either the wild-type *PET117* gene or *GFP* (green fluorescent protein) as a control, both with a C-terminal V5 tag. **a** Expression of the transgenes in the stably transduced control (*C*) and patient (*P*) fibroblasts was verified after SDS-PAGE separation and western blot detection with anti-V5. GFP-V5 and Pet117-V5 are indicated with open and closed arrow heads, respectively. **b** Blue native electrophoreses and western blot analysis of the cell lines. Membranes were probed for complex IV (COX-IV) or complex II (SDHA), the latter as a loading control. **c** The activity of the different respiratory chain complexes and CV were analyzed in three independently obtained mitochondrial extracts from the stably transduced cells. The efficiency of the *PET117* complementation is expressed as percentage of the C + GFP complemented cell line. The control ranges of 109 untransduced control fibroblast lines are indicated. ***p = 0.001 in an 2-way ANOVA. **d** Relative increase in activity of the different enzyme complexes compared between the GFP and *PET117* transduced cell lines of each individual

Complex IV protein expression levels in cultured primary fibroblast cell lines obtained from the patients were compared with those in control cell lines by Blue native (BN)-PAGE separation followed by western blot analysis of the respiratory chain complexes. As shown in Fig. 2, Panel a, the patient fibroblasts had severely reduced amounts of complex IV protein, explaining the reduction in complex IV enzyme activity. The other respiratory chain complexes were present in amounts comparable to those in the control samples, albeit with considerable natural variations as is also apparent in the enzymatic control ranges as shown in Fig. 3, Panel c). Analysis of the complex IV subunits, detected after SDS-PAGE separation followed by western blotting, revealed a severe reduction of the complex IV subunits COX-I, COX-II, and COX-IV in the patient fibroblasts as compared to the levels in a panel of control fibroblasts (Fig. 2, Panel b).

### Genetic analysis of subject 1 and 2

Whole exome sequencing (WES) was used to identify the underlying genetic defect causing the isolated complex IV deficiency. After filtering for common polymorphisms as explained in the materials and methods, we performed a detailed investigation of the variants in the disease genes known to be associated with isolated complex IV deficiency, but this did not reveal any candidate disease causing variants. Subsequently, the genetic variants in the complete WES datasets were compared between the sisters, resulting in three groups of shared variants; compound heterozygous variants, homozygous variants, and variants present in MitoCarta2.0 (Calvo et al. 2016) (shown in Table 2 and Supplementary Table 1). To find the most promising disease candidate gene, we analyzed each of the variants for several criteria, as indicated in Supplementary Table 1 and the “Materials and Methods”. Based on these criteria a single homozygous variant in *PET117* was identified as the only candidate disease causing variant.

**Table 2:** Meta data of WES analysis of subject 1 and subject 2

	Subject 1	Subject 2
# Variants called	128,536	78,497
Applied filters		
dbSNP v.137 (<0.5%)	22,768	8349
In-house database (<0.5%)	12,239	2353
Exonic/splice sites	1214	694
Non-synonymous	779	448
Shared variants		
Compound heterozygous	12	
Homozygous	15	
Mitocarta2.0	8	

A homozygous nonsense mutation in the gene encoding *PET117* (NM_001164811) was detected in both patients. The cytosine to thymine (C>T) mutation at position c.172 results in a premature stop codon at position 58 of the protein. The mutation was confirmed by Sanger sequencing and cosegregated within the family (Fig. 2, Panel c). This variant is not present in genetic variant databases (dbSNP, EVS, ExAC, in-house database).

### Copper treatment of patient cells

Human *PET117* has been identified in a bioinformatics approach to identify human complex IV assembly factors (Szklarczyk et al. 2012). Results from previous studies had indicated that Pet117 may interact with COX17, a mitochondrial copper chaperone for complex IV (Szklarczyk et al. 2012). Therefore, we studied a possible role for Pet117 in the copper insertion into complex IV. For this purpose, we treated the fibroblast cell line from subject 1 with CuCl_2_. The patient fibroblast were treated for 10 days at concentrations of 100 or 200 µM, since these conditions have been shown before to lead to a complete (Salviati et al. 2002) or partial (Baertling et al. 2015) rescue of complex deficiency caused by pathogenic variants in genes encoding proteins involved in copper delivery to complex IV. We performed non-denaturing BN-PAGE using *n*-dodecyl *β*-d-maltoside lysed mitochondrial protein with subsequent immunoblotting, which revealed no positive effect on complex IV levels in Pet117 deficient patient fibroblasts (data not shown).

### Lentiviral complementation

To establish whether the *PET117* mutation as found in the patients indeed is the cause of the complex IV deficiency in the patients fibroblasts, we performed a genetic complementation of the fibroblasts of subject 1 using lentiviral particles that contain the wild *PET117* cDNA with a C-terminal V5-epitope tag. As a control for the procedure we used the gene encoding green fluorescent protein (GFP) also with a C-terminal V5-tag. The expression of the transgene products Pet117-V5 and GFP-V5 was confirmed by SDS-PAGE and western blot analysis of the fibroblasts cell lines (Fig. 3, Panel a). The apparent molecular masses are consistent with the expected masses of the tagged proteins.

Blue native gel analysis of the respiratory chain complexes showed a clear increase in the levels of fully assembled complex IV in the patient cell line complemented with the wild-type *PET117*, as compared to the patient cell line transduced with the GFP-V5 expression construct (Fig. 3, Panel b). Activity measurements of the respiratory chain enzymes in these cell lines showed that the expression of wild-type *PET117* in the fibroblasts of subject 1 resulted in a concomitant specific and significant increase in the activity of complex IV. The activities of the other respiratory chain enzyme complexes were similar to those in the GFP-expressing patient cell line (Fig. 3, Panels c and d). As indicated by the control ranges based on measurements of 109 different control fibroblast lines, the complex IV enzyme activity of the patient fibroblast was restored to control levels (Panel c).

## Discussion

In this paper we describe two sisters with a mitochondrial disease presenting with neurodevelopmental regression and cerebral lesions in the medulla oblongata. Both patients had an isolated deficiency of complex IV of the respiratory chain. WES analysis of the patients revealed a homozygous variant in *PET117* as the most likely cause of the disease, based on the nature of the mutation (nonsense mutation resulting in a premature stop codon), the described mitochondrial localization (Szklarczyk et al. 2012), and the putative involvement in complex IV assembly. We thus report for the first time mutations in *PET117* as a cause for complex IV deficiency.

In addition to the CNS lesions that are present in both patients, in one of the two patients gastrointestinal pathology (PLE) was observed. Electron microscopy revealed increased numbers of mitochondria in intestinal cells of subject 2, which may suggest a mitochondrial involvement, although this may also be a secondary phenomenon due to the intestinal pathology. We considered the possibility of a second mutation in subject 2 as a possible explanation for the intestinal and immunological problems, but a detailed analysis of the WES data, including an analysis of variants that are present in subject 2 but not in subject 1, could not reveal a genetic explanation. As subject 1 does not have these additional symptoms, it remains to be elucidated whether the gastrointestinal and immunological features are part of the mitochondrial disease caused by the *PET117* mutation.

The human *PET117* gene has very weak homology with its yeast counterpart and could only be identified from the human genome by a specialized bioinformatics approach (Szklarczyk et al. 2012). The gene, located on chromosome 20, consists of two exons and encodes for only 81 amino acids. With BLAST searches no homologies with other proteins have been found. Knockout of the yeast *PET117* has initially been described as converting a *petite* phenotype to these cells, pointing to a possible role in mitochondrial function (McEwen et al. 1986, 1993).

The homozygous missense mutation found in the patients results in a premature stop codon. In many cases this does not result in the formation of a truncated protein, but rather leads to nonsense mediated decay. Due to the lack of a specific antiserum to Pet117 we could not analyze the expression of Pet117 in the fibroblast cell lines of these patient. The isolated deficiency of complex IV enzyme activity was accompanied by a specific decrease in holo-complex IV protein levels. The protein expression levels of several individual complex IV subunits were found to be reduced as compared to levels in control fibroblast cell lines. Similar observations have been described for defects in other complex IV assembly factors, such as *C12orf62*/*COX14* (Weraarpachai et al. 2012) and *PET100* (Lim et al. 2014; Olahova et al. 2015). This is probably due to increased turnover of the subunits when the assembly of complex IV is impaired.

Our functional complementation studies of the patient cell lines showed that the complex IV activity and protein levels of the assembled complex were restored to near-normal levels upon transduction with wild-type *PET117* cDNA. This proves that the complex IV deficiency is caused by the *PET117* gene defect and indicates a critical role of Pet117 in the biogenesis of complex IV. Although previous studies suggested a possible role of Pet117 in the copper insertion into complex IV, our data indicate that this is not the case. Very recently, a yeast study demonstrated that Pet117 interacts with Cox15, the heme *a* synthase that is an essential factor for complex IV assembly. It was shown that Pet117 is required for oligomerization of Cox15 (Taylor et al. 2016). Previously, it has been shown that Cox15 forms complexes with Shy1, the yeast homolog of Surf1, and that both proteins form hetero-oligomers and associate with complex IV assembly intermediates and cooperatively insert heme *a* (Bareth et al. 2013). The recent data indicate that Pet117 is an essential factor in the coupling of heme *a* synthesis with complex IV assembly (Taylor et al. 2016). This is compatible with our results, showing that a mutation in this gene caused complex IV deficiency and that exclude a role of Pet117 in copper insertion. For future research, it would be of interest to study the heme levels in samples of our patients in more detail, as was done for the Cox15 patients (Antonicka et al. 2003).

In conclusion, we present two patients with complex IV deficiency caused by mutations in *PET117* and thus present a novel genetic cause for mitochondrial disease.

## Electronic supplementary material

Below is the link to the electronic supplementary material.

Supplementary material 1 (PDF 196 kb)
