# Supplementary material for: Mutated PET117 causes complex IV deficiency and is associated with neurodevelopmental regression and medulla oblongata lesions
Source: Hum Genet. 2017 Apr 6;136(6):759–69. doi: 10.1007/s00439-017-1794-7 (PMC5429353; doi:10.1007/s00439-017-1794-7)
Supplement: Supplementary file 1 — Supplementary material 1 (PDF 196 kb) [file 439_2017_1794_MOESM1_ESM.pdf]

Supplementary Table 1: Whole exome sequencing results.

a shared compound heterozygous variants

Gene name	Chromosome	Genomic position (hg19)	Gene id	variant	zygosity	protein	dbSNP v.137 freq (%)	dbSNP v.137 RS	ExAC (%)	Align	SIFT	Polyphen	phyloP	Grantham Score	OMIM	Disease (based on OMIM)	Selection criteria
ARHGAP31	chr3	g.119120744C>T	NM_020754	c.1145C>T	Hz	p.Ser382Phe	0	rs370215363	0,007	C0	0,02	0,087	1,985	155	610911	Adams-Oliver syndrome 1	a, b
ARHGAP31	chr3	g.119133120C>A	NM_020754	c.2344C>A	Hz	p.Pro782Thr	0,05	rs139659618	0,04	C0	0,30	0,001	0,566	38	610911	Adams-Oliver syndrome 1	a, b
PRUNE2	chr9	g.79318820T>G	NM_015225	c.7709A>C	Hz	p.Glu2570Ala	0,37	rs140137838	0,26	C0	0,38	0,265	0,305	107	610691		a
PRUNE2	chr9	g.79322539T>C	NM_015225	c.4651A>G	Hz	p.Asn1551Asp	0,09	rs186649425	0,04	C0	0,78	0,004	0,124	23	610691		a
ANKK1	chr11	g.113270015G>T	NM_178510	c.1324G>T	Hz	p.Gly442Cys	0	rs4938016	0,23	C0	0,02	0,000	0,274	159	608774	Dopamine receptor D2, reduced brain density of	a
ANKK1	chr11	g.113270478C>T	NM_178510	c.1787C>T	Hz	p.Pro596Leu	0,23	rs7104979	0,27	C65	0,00	0,968	2,659	98	608774	Dopamine receptor D2, reduced brain density of	a
ALDH3B2	chr11	g.67432798C>T	NM_000695	c.664G>A	Hz	p.Val222Met	0,37	rs113012592	0,13	C0	0,04	0,909	2,687	21	601917		a
ALDH3B2	chr11	g.67433023C>T	NM_000695	c.439G>A	Hz	p.Val147Met	0,41	rs148026822	0,13	C0	0,03	0,079	1,777	21	601917		a
OR3A1	chr17	g.3195336A>G	NM_002550	c.541T>C	Hz	p.Tyr181His	0,46	rs61734042	0,27	C0	0,00	0,996	2,201	83	n.a.		d
OR3A1	chr17	g.3195353T>C	NM_002550	c.524A>G	Hz	p.Asn175Ser	0,46	rs61734043	0,27	C0	0,00	0,126	0,285	46	n.a.		a, d
BZRAP1	chr17	g.56386084T>A	NM_004758	c.4549A>T	Hz	p.Thr1517Ser	0,09	rs147085967	0,08	C0	0,97	0,005	0,482	58	610764		a
BZRAP1	chr17	g.56405103C>T	NM_004758	c.179G>A	Hz	p.Ser60Asn	0,09	rs146385838	0,08	C0	0,00	0,679	0,764	46	610764		a

b shared homozygous variants

Gene name	Chromosome	Genomic position (hg19)	Gene id	variant	zygosity	protein	dbSNP v.137 freq (%)	dbSNP v.137 RS	ExAC (%)	Align	SIFT	Polyphen	phyloP	Grantham Score	OMIM	Disease (based on OMIM)	Selection criteria
TTLL10	chr1	g.1115510C>T	NM_001130045	c.296C>T	Ho	p.Pro99Leu	0,23	rs114359609	0,24	C0	0,31	0,001	-0,765	98	n.a.		a
SCNN1D	chr1	g.1222657G>A	NM_001130413	c.1288G>A	Ho	p.Asp430Asn	0	rs140170231	0,31	C0	0,73	0,013	0,270	23	601328		a
SCNN1D	chr1	g.1226311C>T	NM_001130413	c.1954C>T	Ho	p.Pro652Ser	0	rs113608779	0,08	C0	0,42	0,002	-0,155	74	601328		a
ANKRD65	chr1	g.1354495T>C	NM_001145210	c.1185A>G	Ho	p.Ile395Met	0,37	rs141533546	0,38	C0	0,15	0,029	-0,356	10	n.a.		a
ITPKB	chr1	g.226923391C>T	NM_002221	c.1769G>A	Ho	p.Arg590Gln	0	rs774932416	0,003	C0	0,00	0,006	-1,112	43	147522		a
AGRN	chr1	g.981801C>T	NM_198576	c.2936C>T	Ho	p.Pro979Leu	0	rs562644551	0,005	C0	0,03	0,975	0,921	98	103320	Myasthenic syndrome, congenital, with pre- and postsynaptic	a, b
LAMA4	chr6	g.112575200G>T	NM_001105206	c.153C>A	Ho	p.Ser51Arg	0	rs782192817	0,002	C0	0,00	0,001	0,585	110	600133	Cardiomyopathy, dilated, 1JJ	a, b
CSPP1	chr8	g.68074097A>C	NM_024790	c.2575A>C	Ho	p.Ser859Arg	0,14	rs201259375	0,050	C0	0,42	0,018	0,622	110	611654	Joubert syndrome 21	a, b
EVPL	chr17	g.74003216C>T	NM_001988	c.6070G>A	Ho	p.Ala2024Thr	0	rs149512337	0,06	C0	0,10	0,129	1,263	58	601590		a, d
EVPL	chr17	g.74004307C>T	NM_001988	c.4979G>A	Ho	p.Arg1660Gln	0	rs199686324	0,05	C0	0,29	0,571	4,088	43	601590		a, d
EVPL	chr17	g.74004600C>A	NM_001988	c.4686G>T	Ho	p.Glu1562Asp	0	rs113394630	0,07	C0	0,55	0,003	0,409	45	601590		a, d
EVPL	chr17	g.74004928G>A	NM_001988	c.4358C>T	Ho	p.Pro1453Leu	0,05	rs113135904	0,06	C0	0,00	0,989	4,061	98	601590		d
EVPL	chr17	g.74007865G>C	NM_001988	c.2556C>G	Ho	p.Ser852Arg	0	rs141134319	0,06	C0	0,02	0,391	1,734	110	601590		a
PET117	chr20	g.18122927C>T	NM_001164811	c.172C>T	Ho	p.Gln58*	0	-	0,00	n.a.	n.a.	n.a.	4,617	1000	614771		
ASXL1	chr20	g.31023964G>T	NM_015338	c.3449G>T	Ho	p.Gly1150Val	0	rs773823004	0,002	C0	0,34	0,017	0,332	109	612990	Bohring-Opitz syndrome; Myelodysplastic syndrome	a, b

c shared Mitocarta2.0 variants

Gene name	Chromosome	Genomic position (hg19)	Gene id	variant	zygosity	protein	dbSNP v.137 freq (%)	dbSNP v.137 RS	ExAC (%)	Align	SIFT	Polyphen	phyloP	Grantham Score	OMIM	Disease (based on OMIM)	Selection criteria
ALDH18A1	chr10	g.97370014T>C	NM_002860	c.2146A>G	He	p.Ser716Gly	0	-	0	C55	0	0,999	4,619	56	138250		c
HEMK1	chr3	g.50616356A>C	NM_016173	c.769A>C	He	p.Ser257Arg	0	-	0	C0	0,69	0,004	2,582	110	n.a.		a, c
MRPL23	chr11	g.1977627G>C	NM_021134	c.439G>C	He	p.Val147Leu	0,05	rs151012849	0,16	C0	0,30	0,013	0,318	32	600789		a, c
MSRA	chr8	g.9912075C>T	NM_012331	c.49C>T	He	p.Leu17Phe	0	-	0	C0	0,18	0,025	-0,320	22	n.a.		a, c
MTRF1	chr13	g.41827127T>G	NM_004294	c.547A>G	He	p.Asn183Asp	0	-	0	C0	0,37	0,138	2,684	68	604601		a, c
NEU4	chr2	g.242757937C>G	NM_001167599	c.1057C>G	He	p.Arg353Gly	0	rs200439365	0,37	C0	0,42	0,039	-0,201	125	608527		a, c
PET117	chr20	g.18122927C>T	NM_001164811	c.172C>T	Ho	p.Gln58*	0	-	0,00	n.a.	n.a.	n.a.	4,617	1000	614771		
SARS2	chr19	g.39412913A>G	NM_017827	c.364-6T>C	He	p.?	0	-	0	§	n.a.	n.a.	-1,474	0	612804		a, c
